# Supplementary material for: Unexpected large evasion fluxes of carbon dioxide from turbulent streams draining the world’s mountains
Source: Nat Commun. 2019 Oct 25;10:4888. doi: 10.1038/s41467-019-12905-z (PMC6814801; doi:10.1038/s41467-019-12905-z)
Supplement: Supplementary file 1 — Supplementary Information [file 41467_2019_12905_MOESM1_ESM.pdf]

Supplementary Information for

UNEXPECTED LARGE EVASION FLUXES OF CARBON DIOXIDE FROM TURBULENT STREAMS DRAINING THE WORLD'S MOUNTAINS

by ÅSA HORGBY et al.

Supplementary Note 1

Marx and colleagues (2017) show that small streams (catchment sizes <30 km^2) have high geochemical variability but are skewed towards the carbonate end-member. This it related to short residence times in small headwater catchments, which favors fast weathered bedrock, such as carbonate bedrock weathering, rather than silicate weathering[1]. We repeated the analysis from Marx and colleagues using the same database, the Global River Chemistry database (GLORICH)[2], together with data from our Swiss monitoring stations. The geochemical characterization shows that the Swiss sites are indeed influenced by carbonate bedrock weathering (Supplementary Figure 4).

Supplementary Note 2

We evaluated differences between geopredictors for Swiss streams derived from data sets with different resolutions (highly resolved Swiss dataset and lower resolved globally available dataset; Supplementary Figure 6) to be confident that the stream channel slopes (Supplementary Figure 6A) stream altitude (Supplementary Figure 6B) and stream discharge (Supplementary Figure 6C) did not deviate considerably depending on data resolution. The distributions of the data were similar for the two datasets, although slope was somewhat lower in the high-resolution data set (median 0.039 m m^{-1}, CI: 0.004 and 0.176 m m^{-1}) compared to the low-resolution data set (median 0.055 m m^{-1}, CI: 0.004 and 0.222 m m^{-1}). Altitude was slightly higher in the high-resolution data set (median 902 m, CI: 415 and 2185 m) compared to the low-resolution data set (median 834 m, CI: 406 and 2141 m). Discharge was similar between the high-resolution data set (median 0.38 m^3 s^{-1}, CI: 0.09 and 1.77 m^3 s^{-1}) and the low-resolution data set (median 0.38 m^3 s^{-1}, CI: 0.12 and 1.82 m^3 s^{-1}).

Moreover, for all our monitoring stations in the Swiss Aps (Supplementary Figure 1) we measured stream channel slope every 10 meters' distance in the field using a dGPS system. We then compared the difference in stream channel slopes calculated for whole stream reaches with channel slopes calculated from segments of 10 m in length each. We found that in average the slope is underestimated by 0.022 m m^{-1} when using mean reach slope and not considering the variations in slopes along a stream reach. The maximum slope measured along the reach (10 m sub-reaches) deviated significantly from the mean reach slopes, and was up to 0.60 m m^{-1} higher. Across all 12 catchments, the maximum slope was 0.42 m m^{-1} higher compared to the reach slope. This suggests that the k_{CO2} values may be even higher than estimated in this study for Switzerland (median 86.4 m d^{-1}, CI: 6.0 and 461.9 m d^{-1}) and for mountain streams worldwide (median 25.6 m d^{-1}, CI: 3.5 and 410.6 m d^{-1}). Predicted streamwater pCO_2 was similar in Swiss streams (median 705 µatm, CI: 380 and 1224 µatm) and at the global extent (median 737 µatm, CI: 317 and 1644 µatm). 10.8% of the Swiss mountain streams, and the same proportion of the mountain streams worldwide has negative ΔCO_2 meaning that they fall below atmospheric saturation. The median areal CO_2 fluxes are higher from Swiss mountain streams (median 3.6 kg C m^{-2} yr^{-1}, CI: -0.5 and 23.5 kg C m^{-2} yr^{-1}) compared to mountain streams worldwide (median 1.1 kg C m^{-2} yr^{-1}, CI: -0.5 and 32.1 kg C m^{-2} yr^{-1}) (Supplementary Figure 7).

Supplementary Note 3

Estimating CO_2 fluxes based on mean or median values of CO_2 and discharge, instead of highly resolved data, may induce errors due to the temporal variability. We compared CO_2 fluxes derived from measured data at 10-minute time steps with CO_2 fluxes predicted using our CO_2 prediction model combined with Q from GloRiC. Due to data availability, this analysis was possible at 7 of our 12 high-altitude Alpine monitoring stations. Despite high temporal variability in CO_2 fluxes, we found median areal fluxes at the monitoring stations corresponding relatively well to the areal fluxes that we predicted in our study where the slope between F_{CO2} predicted by the model and F_{CO2} calculated from 10-minute time series was -0.921 ± 0.284 (R^2 = 0.68, n = 7, P = 0.0022) (Supplementary Figure 9).

Supplementary Figures

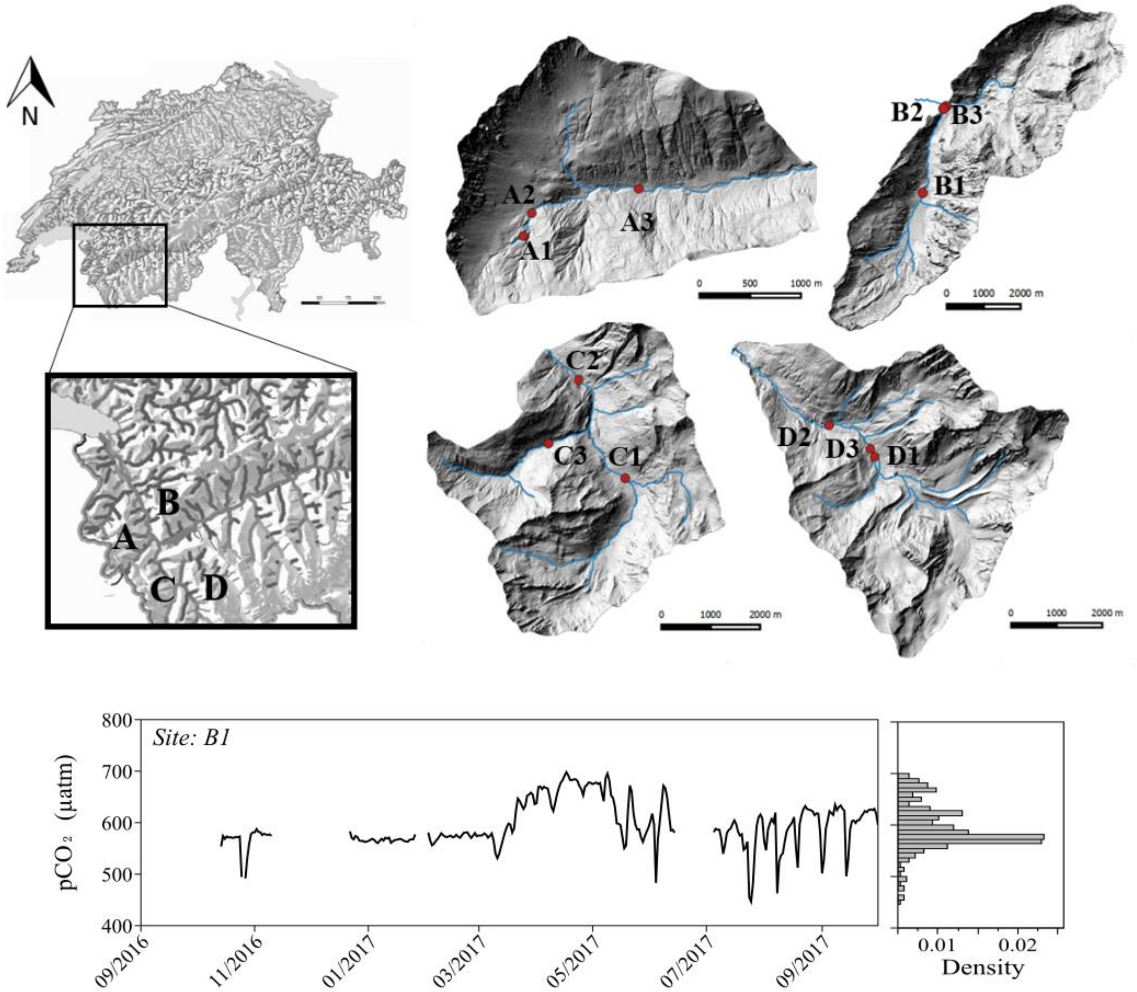

Supplementary Figure 1. We continuously monitored *p*CO_2 in 12 streams located in 4 Alpine catchments in Switzerland. Our 12 mountain stream monitoring stations measured streamwater *p*CO_2 levels close to saturation throughout the year (median *p*CO_2 397 to 673 µatm; Supplementary Table 1).

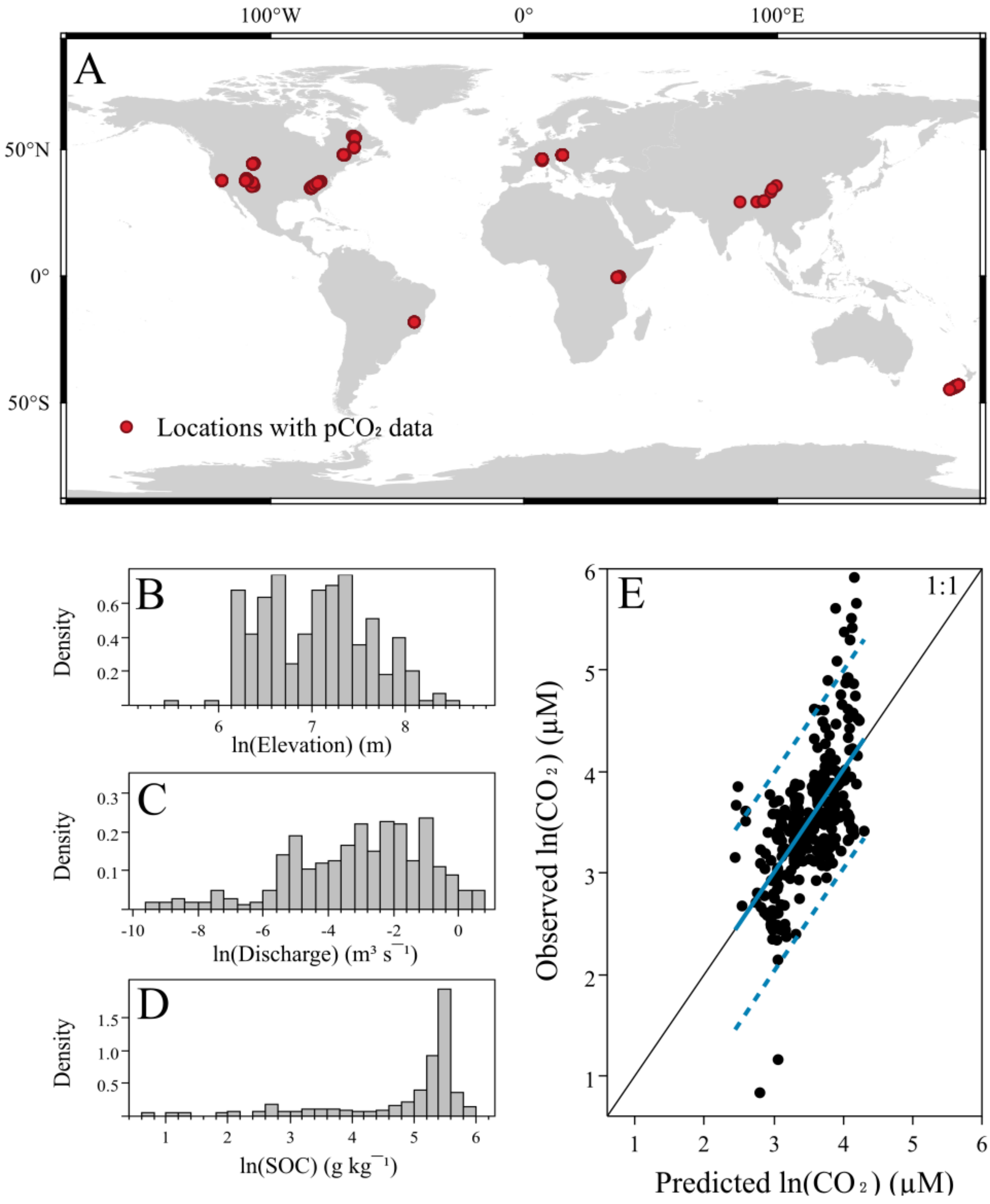

Supplementary Figure 2. Data input of the CO_2 model. Sampling locations for the mountain stream CO_2 data used for the prediction model (A). Shown are also density distributions of elevation (B), discharge (C) and soil organic carbon (SOC) (D), used as input parameters for the prediction of streamwater CO_2 concentrations (E). Observed versus predicted CO_2 followed the 1:1 line (blue) and fell within the 95% prediction confidence intervals (dashed blue lines) except for at very high CO_2 concentrations were CO_2 was underpredicted.

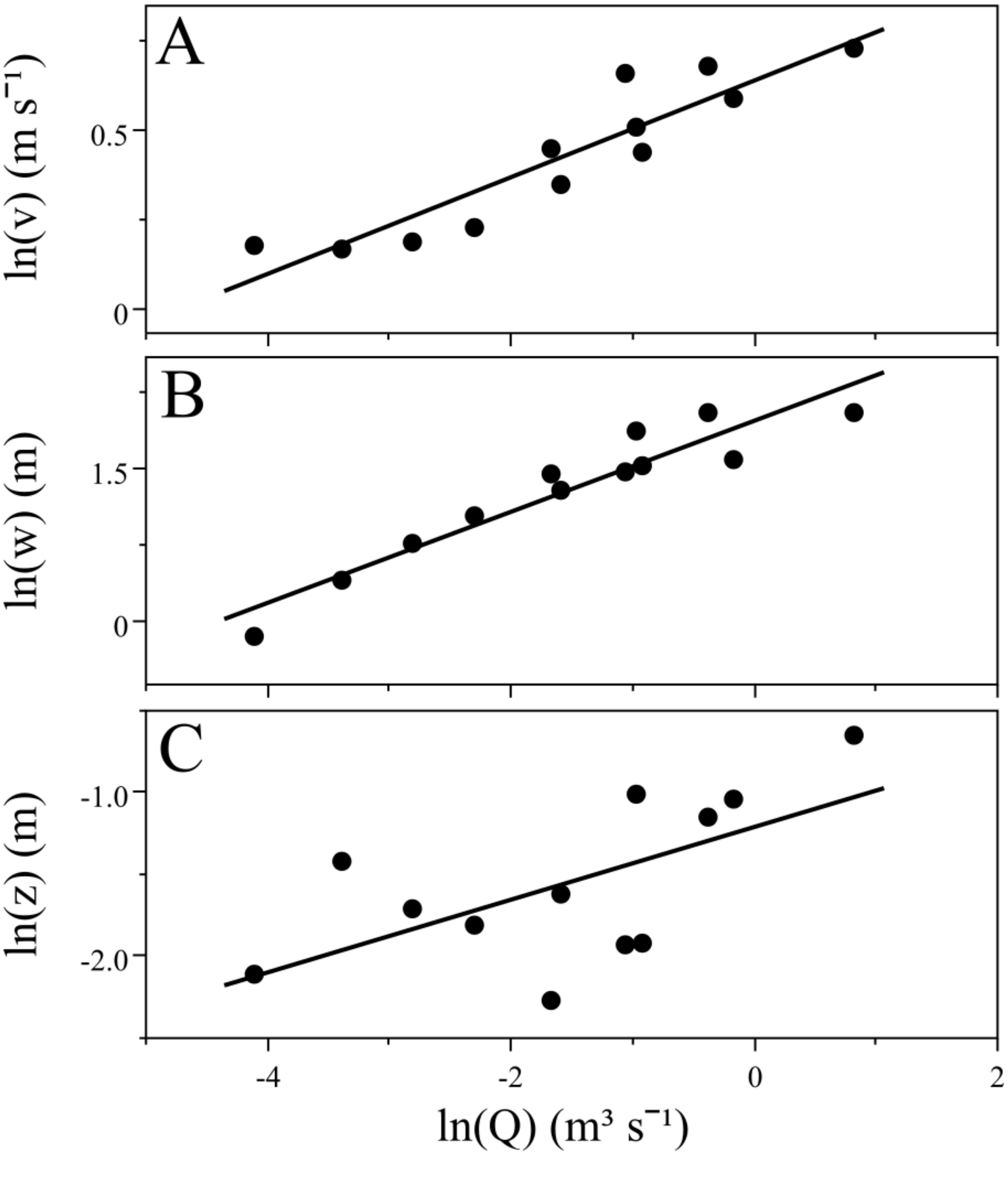

Supplementary Figure 3. Hydraulic scaling relationship (natural log) between annual average stream discharge (Q) and flow velocity (v), stream width (w) and stream depth (z). The relationships are derived from 141 measurements (7-17 measurements per sites) at 12 mountain stream monitoring stations in the Swiss Alps (velocity: $\ln(v) = 0.365 \times \ln(Q) - 0.403$, $R^2 = 0.87$; width: $\ln(w) = 0.447 \times \ln(Q) + 1.961$, $R^2 = 0.90$; depth: $\ln(z) = 0.222 \times \ln(Q) - 1.212$, $R^2 = 0.39$).

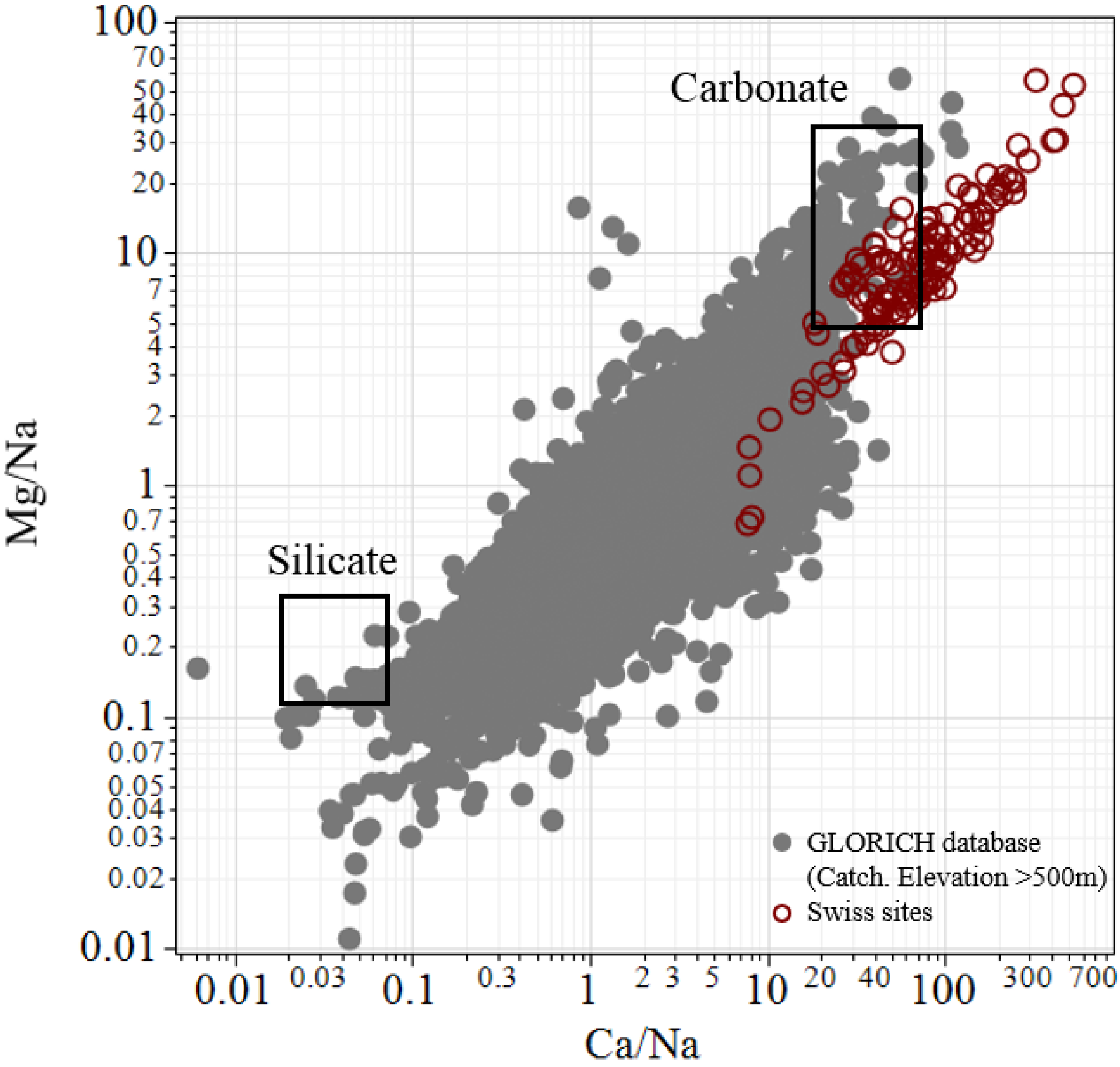

Supplementary Figure 4. Water samples from the Swiss sites (red) are all close to the carbonate end-member in this Na^+-normalized mixing diagram. The Swiss sites have similar Mg^{2+}/Na^+ and Ca^{2+}/Na^+ molar ratios as the carbonated sites in the GLORICH database (gray)[1–3].

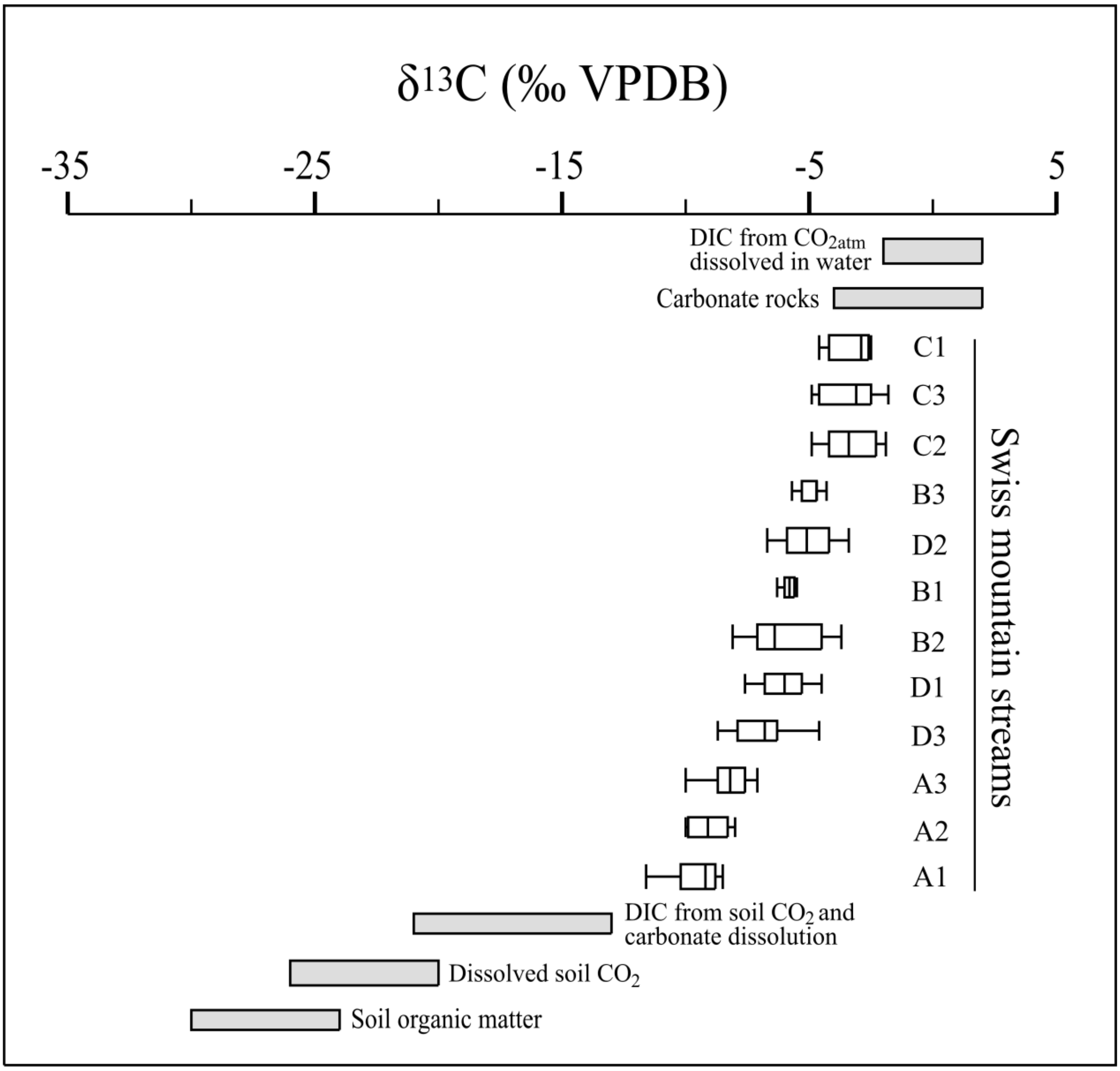

Supplementary Figure 5. Isotopic composition of the streamwater DIC (δ^{13}C-DIC) for the 12 study streams (7 to 15 samples per stream). End-members are adopted from refs. [4,5]. The box plots show median and quartile δ^{13}C-DIC compositions (calculated in JMP 13, SAS Institute Inc., USA).

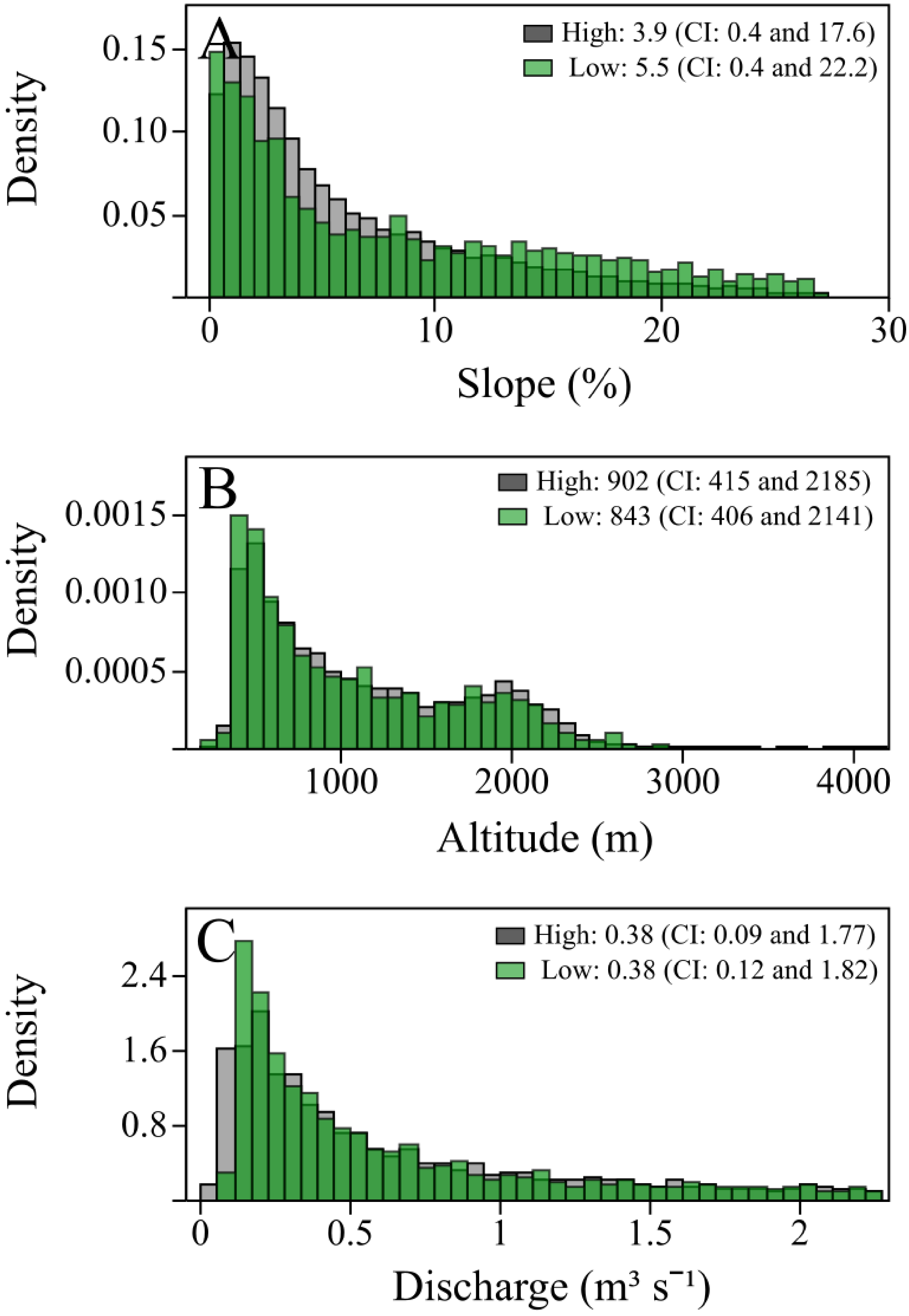

Supplementary Figure 6. Distributions of the main input parameters used for estimation of CO_2 fluxes. Slope (A), altitude (B) and discharge (C) of Swiss mountain streams derived from the high-resolution (grey) and low-resolution (green) data sets.

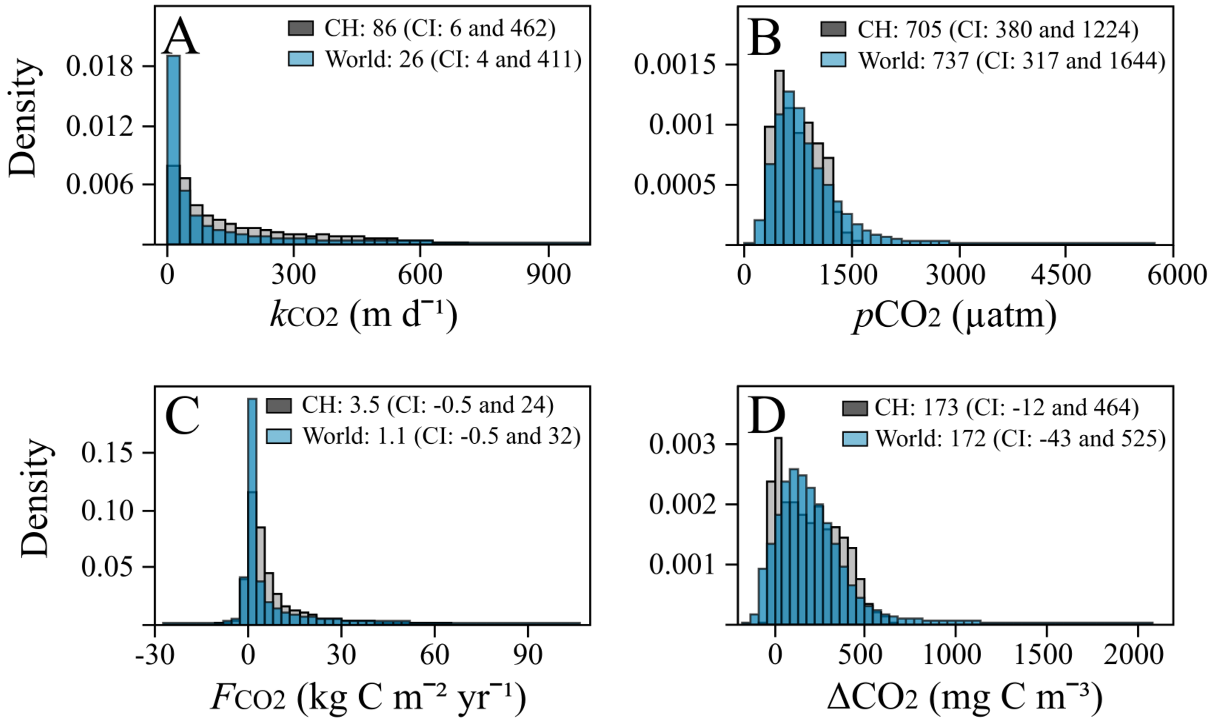

Supplementary Figure 7. Density distributions of k_{CO2} (A), pCO2 (B), F_{CO2} (C) and ΔCO2 (D) calculated for 23,343 Swiss mountain streams (CH; grey) and 1,872,874 mountain streams worldwide (World; blue).

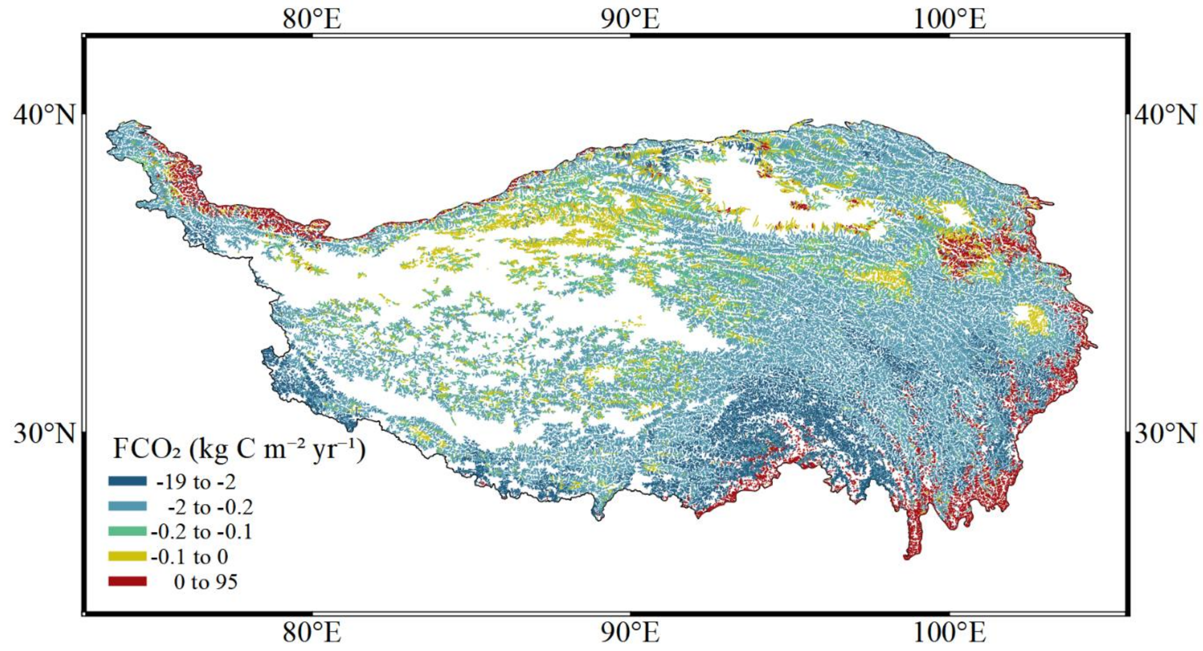

Supplementary Figure 8. Most streams at the Tibetan plateau are undersaturated in CO_2 with respect to the atmosphere and likely acts as a CO_2 sink. Streamwater pCO_2 was mainly undersaturated with respect to the atmosphere (median 288 µatm, CI: 194 and 449 µatm) and 88% of the streams had negative CO_2 fluxes.

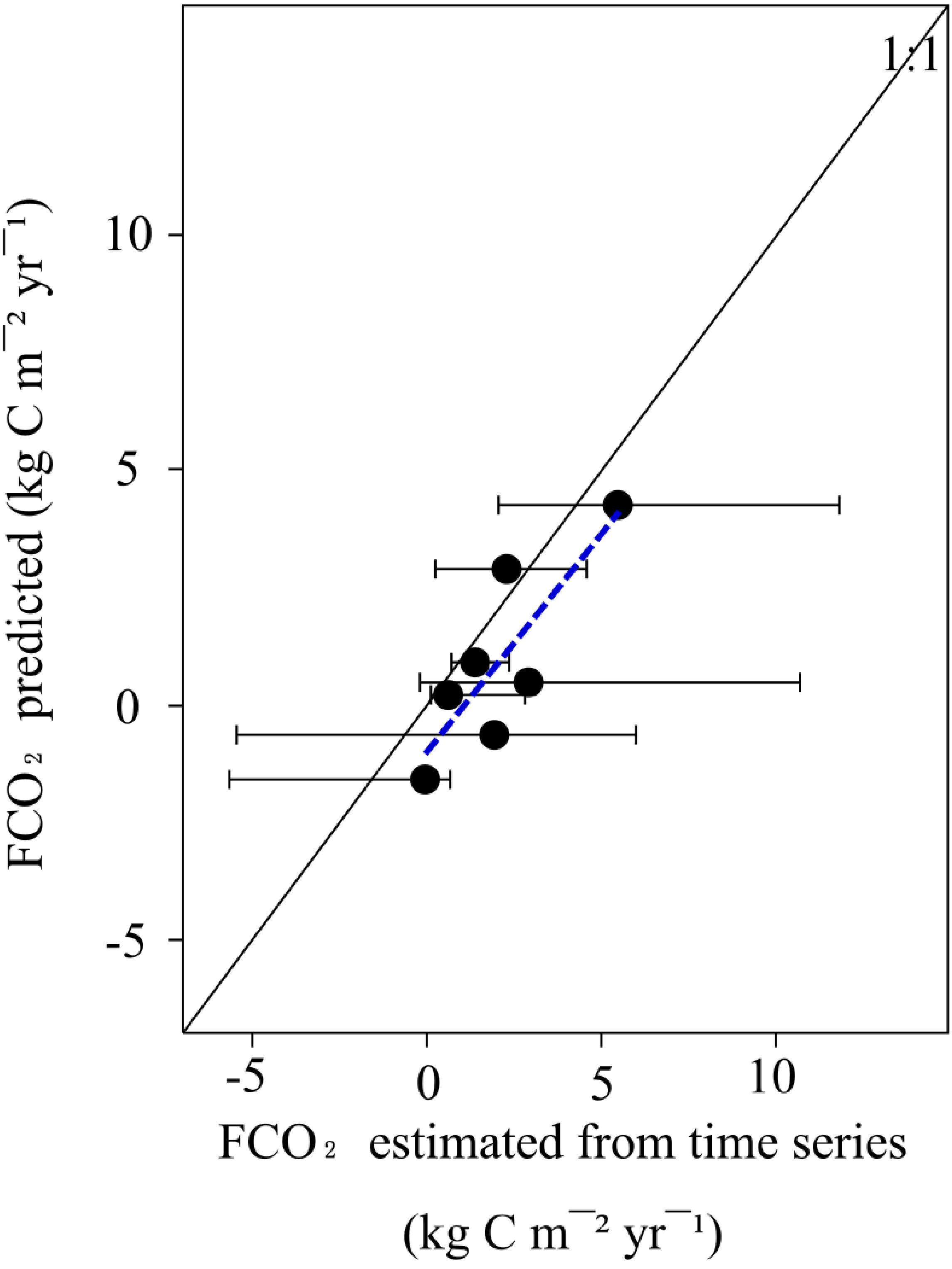

Supplementary Figure 9. The predicted CO_2 flux corresponded well to the median CO_2 flux estimated from time series (10-minute time steps). Median values (black dots) and 95% confidence intervals (error bars) of F_{CO2} predicted from the CO_2 model versus median values based on time series (Predicted $F_{CO2} = 0.926 \pm 0.284 \times$ Measured $F_{CO2} - 0.981 \pm 0.752$, $R^2 = 0.68$, n = 7, P=0.022). Units are expressed in kg C m^{-2} yr^{-1} for consistency with other flux estimates reported in this study.

Supplementary Tables

Supplementary Table 1. Stream characteristics at the 12 Alpine monitoring stations.

Site	Latitude	Longitude	Altitude (m)	Slope (m m-1)	Mean (±std.) *p*CO_2 (μatm)	Median *p*CO_2 (μatm)
A1	46.1549	6.8002	1689	0.056	511±62	502
A2	46.157	6.8012	1630	0.16	527±84	501
A3	46.1593	6.81473	1415	0.097	687±221	593
B1	46.2316	7.10197	1465	0.048	566±69	579
B2	46.2534	7.10963	1201	0.137	457±26	454
B3	46.2535	7.11011	1200	0.138	486±43	473
C1	45.8831	7.13095	1995	0.054	699±220	673
C2	45.9051	7.1156	1774	0.033	472±57	454
C3	45.8937	7.10797	2027	0.059	525±55	523
D1	45.9295	7.24458	2148	0.059	388±43	397
D2	45.935	7.2269	1937	0.103	427±39	430
D3	45.5568	7.14752	2161	0.078	473±132	465

Supplementary Table 2. Groundwater *p*CO_2 data, sampled adjacent to mountain streams in Switzerland.

Catchment	Latitude	Longitude	Groundwater *p*CO_2 (µatm)	Reference
A	46.2305	7.10042	748	Åsa Horgby, *unpublished data*
A	46.2296	7.10156	1789	Åsa Horgby, *unpublished data*
A	46.2296	7.10156	976	Andrea Popp, James Thornton, *personal communication*
A	46.2296	7.10152	245	Andrea Popp, James Thornton, *personal communication*
A	46.2295	7.1012	1936	Andrea Popp, James Thornton, *personal communication*
A	46.2305	7.10042	1729	Andrea Popp, James Thornton, *personal communication*
A	46.2305	7.10042	1362	Andrea Popp, James Thornton, *personal communication*
A	46.2301	7.10035	1343	Andrea Popp, James Thornton, *personal communication*
A	46.2301	7.10035	1072	Andrea Popp, James Thornton, *personal communication*
B	45.8831	7.13095	6303	Lluís Gómez Gener, *personal communication*
B	45.8831	7.13095	2230	Lluís Gómez Gener, *personal communication*

Supplementary References

1. Marx, A. et al. A review of CO_2 and associated carbon dynamics in headwater streams: a global perspective. *Rev. Geophys*. **55**, 560–585 (2017)
2. Hartmann, J., Lauerwald, R. & Moosdorf, N. A brief overview of the GLObal RIver Chemistry Database, GLORICH. *Procedia Earth Planet. Sci.* **10**, 23–27 (2014).
3. Gaillardet, J., Dupré, B., Louvat, P. & Allègre, C. J. Global silicate weathering and CO_2 consumption rates deduced from the chemistry of large rivers. *Chem. Geol.* **159**, 3–30 (1999).
4. Clark, I. D. & Fritz, P. *Environmental isotopes in hydrogeology* (Lewis Publishers, New York, USA, 1997).
5. Pawellek, F. & Veizer, J. Carbon cycle in the upper Danube and its tributaries: $\delta^{13}C_{DIC}$ constraints. *Isr. J. Earth Sci*. **43**, 187–194 (1994).
